# P-826. Diagnostic Yield of Repeat Blood Cultures in HSCT Transplant Patients with Neutropenic Fever at the NIH Clinical Center

**DOI:** 10.1093/ofid/ofaf695.1034

**Published:** 2026-01-11

**Authors:** Shanan Immel, Bickey Chang, Viviane Callier, Zonghui Hu, Gloria Oshegbo, Andrea Beri, Veronique Nussenblatt, Jennifer Cuellar-Rodriguez

**Affiliations:** Division of Intramural Research, National Institutes of Allergy and Infectious Diseases, National Institutes of Health, Washingont, DC; NIAID, Bethesda, Maryland; Frederick National Laboratory for Cancer Research, Bethesda, Maryland; National Institute of Allergy and Infectious Disease, Bethesda, Maryland; National Institute of Health Clinical Center, Bethesda, Maryland; National Institute of Health Clinical Center, Bethesda, Maryland; Division of Intramural Research, National Institutes of Allergy and Infectious Diseases, National Institutes of Health, Washingont, DC; National Institute of Allergy and infectious Diseases, Bethesda, MD

## Abstract

**Background:**

Hematopoietic cell transplant (HCT) is a well-established therapy to treat a number of malignant and non-malignant conditions. Infectious complications are a major cause of morbidity and mortality in the peri-transplant period, and neutropenia is one of the most common underlying conditions leading to infection. The practice of obtaining daily blood cultures on patients with ongoing fever and neutropenia is not uncommon, mostly driven by the transplant team. The Infectious Disease Society of America (IDSA) does not recommend daily blood cultures beyond the first 72 hours of febrile neutropenia unless there is a clinical change in the patient as the yield after that period is low.

**Figure:**
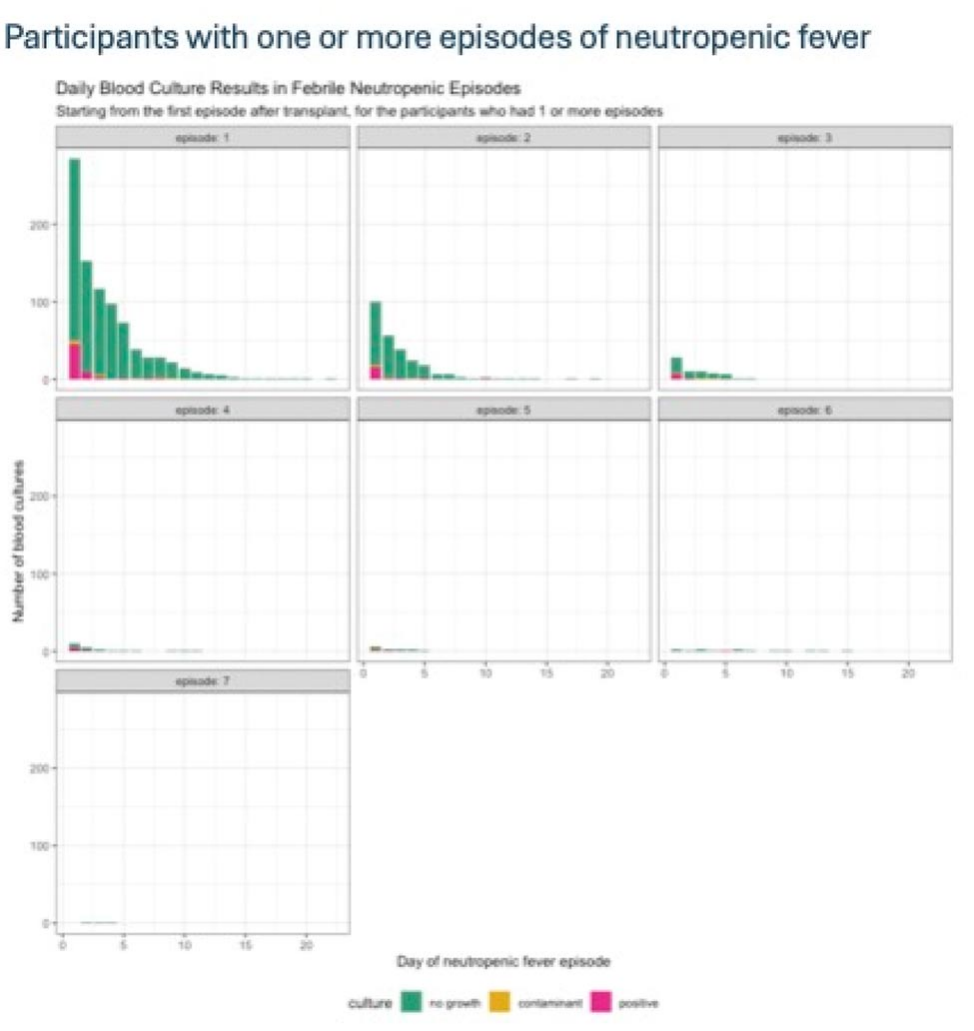
Neutropenic Fever Episodes and Blood Culture Results Consecutive episodes of neutropenic fevers shown here with positive blood cultures (red), negative blood cultures (green), and cultures considered to be contaminants (yellow).

**Methods:**

We performed a retrospective chart analysis of all patients at the National Institutes of Health Clinical Center who received HCT , between January of 2020 to June 2024 and developed febrile neutropenia within 60 days after transplant.

**Results:**

A total of 322 patients met inclusion criteria and had one or more episode(s) of neutropenic fever after transplant. We found that during the first episodes of neutropenic fever the yield of blood cultures on the day 1 was 15.8% (45/284) positive. The diagnostic yield dropped precipitously in subsequent days to 5.9% (9/153), 2.6% (3/117), and 0% (0/97) on days 2, 3, and 4 respectively. A similar pattern was seen in blood cultures obtained in subsequent neutropenic fever episodes. The most common causes of bacteremia in the neutropenic period were *Klebsiella pneumoniae, Streptococcus mitis, Staphylococcus aureus,* and *Escherichia coli.*

**Conclusion:**

Our findings are consistent with previous literature and IDSA guidelines that repeating daily blood cultures for beyond 72 hours into febrile neutropenic episodes has limited diagnostic utility in the absence of clinical change to support additional cultures. These findings may improve diagnostic stewardship, especially in light of recent blood culture bottle shortages.

**Disclosures:**

Veronique Nussenblatt, MD, ScM, MHS, AOA Dx: Stocks/Bonds (Private Company)|Apero Health: Stocks/Bonds (Private Company)|Biotx.ai: Stocks/Bonds (Private Company)|Bluumbio: Stocks/Bonds (Private Company)|ExcepGen: Stocks/Bonds (Private Company)|Granza Bio: Stocks/Bonds (Private Company)|Probably Genetic: Stocks/Bonds (Private Company)|Rancho Stana Fe Bio: Stocks/Bonds (Private Company)|Valink Therapeutics: Stocks/Bonds (Private Company)

